# Survival Benefit of Adding Chemotherapy to Intensity Modulated Radiation in Patients with Locoregionally Advanced Nasopharyngeal Carcinoma

**DOI:** 10.1371/journal.pone.0056208

**Published:** 2013-02-18

**Authors:** Xuemei Ji, Conghua Xie, Desheng Hu, Xia Fan, Yajuan Zhou, Yingjie Zheng

**Affiliations:** 1 Department of Radiation Oncology, Beijing Chaoyang Hospital, Capital Medical University, Beijing, China; 2 Department of Radiation and Medical Oncology, Zhongnan Hospital, Wuhan University, Wuhan, China; 3 Department of Radiation Oncology, Tumor Hosptal of Hubei Province, Wuhan, China; Indiana University School of Medicine, United States of America

## Abstract

**Background:**

To evaluate the contribution of chemotherapy for patients with locoregionally advanced nasopharyngeal carcinoma (NPC) treated by intensity modulated radiotherapy (IMRT) and to identify the optimal combination treatment strategy.

**Patients and Methods:**

Between 2006 and 2010, 276 patients with stage II-IVb NPC were treated by IMRT alone or IMRT plus chemotherapy. Cisplatin-based chemotherapy included neoadjuvant or concurrent, or neoadjuvant plus concurrent protocols. The IMRT alone and chemoradiotherapy groups were well-matched for prognostic factors, except N stage, with more advanced NPC in the chemoradiotherapy arm.

**Results:**

With a mean follow-up of 33.8 months, the 3-year actuarial rates of overall survival (OS), metastasis-free survival (MFS), relapse-free survival (RFS), and disease-free survival (DFS) were 90.3%, 84.2%, 80.3%, and 69.2% for all of the patients, respectively. Compared with the IMRT alone arm, patients treated by concurrent chemoradiotherapy had a significantly better DFS (HR = 2.64; 95% CI, 1.12−6.22; P = 0.03), patients with neoadjuvant-concurrent chemoradiotherapy had a significant improvement in RFS and DFS (HR = 4.03; 95% CI, 1.35−12.05; P = 0.01 and HR = 2.43; 95% CI, 1.09−5.44; P = 0.03), neoadjuvant chemoradiotherapy provided no significant benefit in OS, MFS, RFS, and DFS. Stage group and alcohol consumption were prognostic factors for OS and N stage was a significant predictor for DFS.

**Conclusions:**

Addition of concurrent or neoadjuvant-concurrent chemotherapy to IMRT is available to prolong RFS or DFS for locoregionally advanced NPC. Such work could be helpful to guide effective individualized therapy.

## Introduction

Nasopharyngeal carcinoma (NPC), a relatively rare cancer in most contraries and regions worldwide, is endemic in China, being the eighth most common cause of cancer mortality in China and accounting for 1.45 deaths per 100,000 Chinese people annually [1]. The incidence-to-mortality rate ratios of NPC vary according to different countries, with China having a higher incidence relative to mortality than other populations [2], which might be attributed to distinct treatment regimens, various carcinogenetic factors, and the histologic distribution of NPC in diverse regions.

Radiotherapy (RT) to the nasopharynx, with elective radiation to the neck, used to be the standard treatment for all stages of NPC. Intensity modulated radiotherapy (IMRT), which delivers a high dose of radiation to the tumor while keeping a reduced dose to normal tissues surrounding the NPC region and excellent tumor coverage, has become widely accepted for the treatment of NPC, and has been shown to be more advanced than the conventional two-dimensional technique and three-dimensional conformal radiation in local or regional control. [3] IMRT is target specific (sparingcritical non-target organs) and yields superior treatment outcomes in patients with NPC compared to the conventional treatments mentioned above [4], [5], [6], [7]. Locoregional control has been improved with IMRT, but distant metastasis is the main cause of treatment failure and death [8]. Chemotherapy is important to control distant metastasis of chemoradiosensitive NPC, and thus should play an important role in the treatment of NPC. Alternatively, considering that patients with advanced stage tumors have been shown to have a relatively poor prognosis when treated with IMRT alone [6] and cisplatin-based concurrent chemotherapy has been shown to provide significant survival benefit for patients with locoregionally advanced NPC treated with conventional radiotherapy [9], the addition of chemotherapy to IMRT has been suggested in an attempt to reduce failures and prolong survival. However, the most effective combination of chemotherapy and IMRT has not been well-established. Three retrospective studies have evaluated the contribution of chemotherapy for patients with NPC treated by IMRT and revealed no significant improvement in metastasis-free survival (MFS), disease-free survival (DFS), and overall survival (OS) [10], [11], [12], which were distinct from the efficacy of adding chemotherapy to conventional radiation [9]. The conflicting results might be attributable to basic differences in the radiation technique and confounding factors of NPC treatment in the various patient populations.

We followed a cohort of NPC patients to compare OS, DFS, MFS, and relapse-free survival (RFS) between IMRT alone and IMRT combined with concurrent or neoadjuvant chemotherapy or both to determine the efficacy of chemotherapy and the most effective combined strategy of chemotherapy and IMRT for patients with locoregionally advanced NPC. We also identified the prognostic factors which might affect the prognosis and predict the outcome of patient with locoregionally advanced NPC. Such work could be helpful to achieve an improvement in survival for patients with locoregionally advanced NPC.

## Patients and Methods

### Clinical Data

The current retrospective cohort is composed of 276 newly diagnosed and previously untreated patients with histopathologically-confirmed NPC treated with IMRT at a Hubei Cancer Center in Wuhan, China between January 2006 and December 2010. All patients were Chinese Han. Patients with stage I NPC, had known distant metastases at the time of diagnosis of NPC or were treated with adjuvant chemotherapy, and were excluded from the current study. Patients, alive with <1 year of follow-up were also excluded from this study. Human participant approval was obtained from the ethical committee of Zhongnan Hospital of Wuhan University.

We reviewed the medical records regarding demographic data (age, gender, cigarette smoking, alcohol use, and family history), and clinical characteristics (histologic type, clinical presentation, cancer stage, medical history, physical examination findings, and treatment). According to the World Health Organization (WHO) criteria, NPC was classified into three categories based on the histologic type: type I, squamous cell carcinoma; type II, non-keratinizing carcinoma; and type III, undifferentiated carcinoma [13]. Patients were followed until the date of death or the last contact date, which was 31 May 2012. Response to treatment, survival, pattern of relapse, and distant metastasis were obtained through telephone interviews and follow-up examinations, which included a chest X-ray, abdominal sonography, a whole-body bone scan, and computed tomography or magnetic resonance imaging of the head and neck.

### Treatment

All patients underwent IMRT, with a total radiation dose of 70–74 Gy delivered to gross disease in the nasopharynx, a dose of 60–66 Gy delivered to positive lymph node areas, and a dose of 50–56 Gy to the low-risk local area, in 30–33 fractions, in approximately 7–8 weeks. Clinical target volumes were designed and planning target volumes expanded as per the RTOG 0225 protocol. Doses were modified according to the cancer stage and the size of the positive regional lymph nodes. Irradiation was performed primarily using 6 MV photons and electrons from linear accelerators.

Cisplatin-based chemotherapy, with various sequences and regimens, was added to IMRT in the chemotadiotherapy group. Concurrent chemotherapy was given to 47 patients, mainly with 75–100 mg/m^2^ on day 1 every 3 weeks. A total of 93 patients received neoadjuvant chemotherapy, primarily consisted of 2–3 cycles of DF (cisplatin [25 mg/m^2^ on day 1–3] and 5-fluorouracil [1000 mg/m^2^ on days 1–5]/Tegafur 1000 mg on days 1–5 every 3 weeks) or TP regimen (cisplatin [25 mg/m^2^ on day 1, 2, 3] and docetaxel [75 mg/m^2^ on day 1 every 3 weeks]). Ninety-six patients received neoadjuvant plus concurrent chemotherapy. Among the patients who received neoadjuvant chemotherapy or neoadjuvant plus concurrent chemotherapy, 149 patients were treated with DF regimen, 28 with TP regimen, and 12 with mixed regimens including DF and/or TP regimens and/or other regimen.

### Statistical Analysis

Descriptive statistical analyses were performed to characterize the patients in the IMRT alone and IMRT plus chemotherapy groups. The differences in the distribution of selected demographic variables (age, gender, smoking status, alcohol status, family history of cancer) and clinical characteristics (histologic types, cancer stage group, T stage, N stage, and treatment) were evaluated using a χ^2^ test.

Actuarial rates of OS, DFS, MFS, and RFS were calculated by the Kaplan-Meier method. The primary endpoint for OS, DFS, MFS, and RFS was death, occurrence of relapse or distant metastasis, distant metastasis occurrence, and relapse occurrence of local or nodal tumors, respectively. The time-to-event was calculated from the date of therapy for newly diagnosed NPC to the date-of-event occurrence. Patients who were not known to have an event at the date of last contact or who were lost to follow-up were censored for OS, DFS, MFS, and RFS. Moreover, patients who died during follow-up without an occurrence of the event were censored for DFS, MFS, and RFS. To balance the distribution bias, a multivariate Cox model was built using the demographic variables and clinical characteristics. Associations were quantified using hazard ratios and the 95% confidence intervals. Statistical significance was set at a *P*<0.05. SAS version 9.1 was used to perform all statistical analyses.

## Results

### Patient Characteristics

The mean duration of follow-up was 33.8 months for all patients (33.8±14.6) and 35.1 months for survivors (35.1±13.8). In our cohort study, the median interval was 20 months for occurrence of distant metastasis, 22 months for occurrence of relapse, and 23 months for death before the end of the study. The characteristics of the 276 patients, including 236 patients treated by IMRT combined with neoadjuvant or concurrent chemotherapy or both and 40 patients treated by IMRT alone, are summarized in **Table 1**. The mean age at the time of diagnosis of NPC was 47.1±11.4 years in our cohort study. The distribution of patients was well-balanced among the two groups by major potential prognostic factors, such as the age at the time of diagnosis, gender, cigarette smoking, alcohol use, family history, histologic type, stage group, and T stage (P = 0.092 for age, 0.293 for gender, 0.503 for cigarette smoking, 0.693 for alcohol use, 0.268 for family history, 0.180 for histologic type, 0.470 for stage group, and 0.169 for T stage). Patients treated by combined treatment were more advanced in N stage than patients on IMRT alone (P = 0.047). Therefore, prognostic factors, such as age, gender, cigarette smoking, alcohol use, family history, histologic type, and stage group were further used for building a multivariate Cox model and adjusting the final results.

**Table 1:** Characteristics of patients treated with IMRT alone versus combination of IMRT and chemotherapy.

	IMRT (n = 40)	IMRT plus chemotherapy (n = 236)	
	Number (%)	Number (%)	*P* *
Ages (Years)			
≤50 y	20 (50.0)	151 (64.0)	
>50y	20 (50.0)	85 (36.0)	0.092
Gender			
Female	8 (20.0)	66 (28.0)	
Male	32 (80.0)	170 (72.0)	0.293
Cigarette smoking			
Never	25 (62.5)	136 (57.6)	
Ever	15 (37.5)	100 (42.4)	0.563
Alcohol use			
Never	29 (72.5)	178 (75.4)	
Ever	11 (27.5)	58 (24.6)	0.693
Family history			
Yes	5 (12.5)	47 (19.9)	
No	35 (87.5)	189 (80.1)	0.268
WHO Histology			
I	4 (10.0)	13 (5.5)	
II	23 (57.5)	112 (47.5)	
III	13 (32.5)	111 (47.0)	0.180
Stage group			
II	8 (20.0)	35 (14.8)	
III	22 (55.0)	121 (51.3)	
IV	10 (25.0)	80 (33.9)	0.470
T stage			
T1	4 (10.0)	11 (4.7)	
T2	9 (22.5)	81 (34.3)	
T3	18 (45.0)	78 (33.0)	
T4	9 (22.5)	66 (28.0)	0.169
N stage			
N0	6 (15.0)	19 (8.0)	
N1	19 (47.5)	74 (31.4)	
N2	14 (35.0)	125 (53.0)	
N3	1 (2.5)	18 (7.6)	0.047

* Two-sided χ2 test.

### Survival

The 3-year actuarial rates for OS, MFS, RFS, and DFS were 90.3%, 84.2%, 80.3%, and 69.2% for all of the patients, respectively. The overall mortality rate, distant metastasis rate, relapse rate and rate of patients with relapse or metastasis or both, along with the association between the survivals and treatment for IMRT alone and the addition of chemotherapy to the IMRT arm are shown in **Table 2**. A significant difference was noted in the RFS for patients with or without the addition of chemotherapy (log-rank P = 0.041), with a 3-year RFS rate of 82.0% for chemotherapy plus IMRT and 68.6% for IMRT alone. Patients treated by IMRT alone were at an approximately 2-fold increased risk for occurrence of relapse (HR = 2.13; 95% CI, 1.03−4.40) compared to patients with combination chemotherapy and IMRT. We also showed that freedom from relapse or metastasis or both was 71.1% in the IMRT combined chemotherapy arm and 57.6% in the IMRT alone arm at 3 years, and the survival curves were borderline significantly different (log-rank P = 0.056). A trend toward improved MFS favoring the addition of the chemotherapy arm was observed with a 3-year RFS rate of 84.7% for patients in the combined treatment arm and 81.7% for patients in the IMRT alone arm, but this difference was not significant (HR = 1.78; 95% CI, 0.77−4.12). The OS were not statistically different between the two groups (log-rank P = 0.829).

**Table 2:** Patterns of disease failure in patients treated with IMRT alone versus combination of IMRT and chemotherapy.

	IMRT plus chemotherapy (n = 236)		IMRT (n = 40)				
Failure Pattern	Failure No.	Failure Rate	Failure No.	Failure Rate	Log rank *P*	HR (95%CI)*	*P* *
Locoregional only	41	14.4	11	27.5	0.04	2.13 (1.03–4.40)	0.04
Distant metastases only	33	14.0	8	20.0	0.28	1.78 (0.77–4.12)	0.18
Locoregional or distantmetastases or both	69	29.2	16	40.0	0.06	1.80 (0.10–3.28)	0.05
Death	28	11.9	4	10.0	0.83	1.05 (0.35–3.16 )	0.93

* Adjusted for age, gender, cigarette smoking, alcohol use, family history, histologic types, stage group, T stage and N stage in multivariate Cox model.

**Figure 1:**
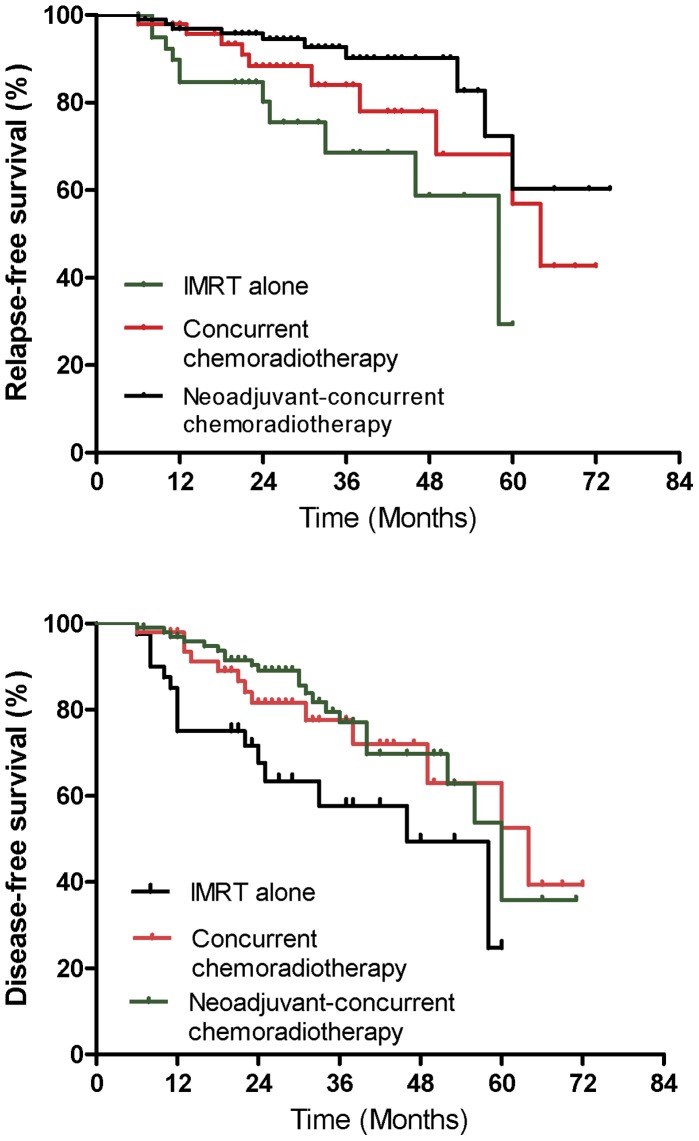
Kaplan-Meier estimates of relapse-free survival and disease-free survival for patients receiving Neoadjuvant-concurrent chemoradiotherapy versus Concurrent chemoradiotherapy versus IMRT alone.

To determine which combined strategy could provide the greatest additional survival benefit, we compared survival among patients treated with IMRT alone, concurrent chemoradiotherapy, neoadjuvant chemoradiotherapy, and neoadjuvant-concurrent chemoradiotherapy (**Table 3**). Patients treated with concurrent chemoradiotherapy had a significantly better DFS (HR = 2.64; 95% CI, 1.12−6.22; P = 0.03) and a borderline significantly better MFS (HR = 2.98; 95% CI, 0.88−10.10; P = 0.08) than patients treated with IMRT alone (**Fig. 1**). The 3-year OS and RFS were 90.7% and 84.0% for the concurrent chemoradiotherapy group, and 94.7% and 68.6% for the IMRT alone group, respectively, with no significant difference between the two groups (P = 0.25 for OS and P = 0.11 for RFS). Compared with the IMRT alone arm, patients treated with neoadjuvant-concurrent chemoradiotherapy had a similar OS and MFS (P = 0.24 and P = 0.53, respectively), and a significantly better RFS and DFS (HR = 4.03; 95% CI, 1.35−12.05; P = 0.01 for RFS and HR = 2.43; 95% CI, 1.09−5.44; P = 0.03 for DFS; **Fig. 1**). Compared with the concurrent chemoradiotherapy arm, neoadjuvant chemotherapy plus concurrent chemoradiotherapy provided no significant benefit in OS, MFS, and DFS. However, patients in the neoadjuvant-concurrent chemoradiotherapy arm had a borderline significant improvement in RFS (HR = 2.44; 95% CI, 0.90−6.67; P = 0.08). There was no statistically significant difference in the OS, MFS, RFS, and DFS between the patients receiving neoadjuvant chemotherapy plus IMRT and patients in the IMRT alone arm. To validate the beneficial effectsderived from adding the concurrent chemotherapy arm or neoadjuvant-concurrent chemotherapy arm, we also analyzed the data in certain stage group or N stage, including stage III, IV, N1, and N2, in which there are analyzable numbers and events. We did find that concurrent chemoradiotherapy provided a borderline significantly better DFS for patients with stage III (HR = 4.04; 95% CI, 0.96−16.96; P = 0.06) and significantly improvement of DFS for patients with stage IV (HR = 46.02; 95% CI, 2.94−720.98; P = 0.006). Neoadjuvant-concurrent chemoradiotherapy contributed to a borderline significantly better RFS (HR = 8.56; 95% CI, 0.86−85.34; P = 0.07) and a significantly better DFS (HR = 7.49; 95% CI, 1.76−31.77; P = 0.006)for patients with stage IV, and a significantly better RFS and DFS for patients with stage N1 (HR = 7.35; 95% CI, 1.58−34.08; P = 0.01for RFS and HR = 4.33; 95% CI, 1.27−14.81; P = 0.02 for DFS).

**Table 3:** Comparison of survival in patients treated with different treatment strategies.

	Overall survival		Distant metastasis-freesurvival		Relapse-free survival		Disease-free survival	
	HR (95%CI)*	*P* *	HR (95%CI)*	*P* *	HR (95%CI)*	*P* *	HR (95%CI)*	*P* *
Concurrent chemoradiotherapy vs IMRT alone	2.48 (0.53–11.68)	0.25	2.98 (0.88–10.10)	0.08	2.23 (0.81–6.13)	0.11	2.64 (1.12–6.22)	0.03
Neoadjuvant-concurrent chemoradiotherapy vs IMRT alone	2.55 (0.54–12.04)	0.24	1.39 (0.49–3.92)	0.53	4.03 (1.35–12.05)	0.01	2.43 (1.09–5.44)	0.03
Neoadjuvent chemoradiotherapy vs IMRT alone	0.286 (0.07–1.10)	0.07	1.62 (0.60–4.40)	0.34	1.62 (0.69–3.80)	0.27	1.38 (0.70–2.75)	0.35
Neoadjuvant-concurrent chemoradiotherapy vs Concurrent chemoradiotherapy	1.64 (0.46–5.82)	0.44	1.02 (0.31−3.33)	0.98	2.44 (0.90−6.67)	0.08	1.23 (0.58−2.60)	0.59

* Adjusted for age, gender, cigarette smoking, alcohol use, family history, histologic types, stage group, T stage and N stage in multivariate Cox model.

In order to further verify the contribution of combining chemotherapy with IMRT and eliminate the potential impact from confounding chemotherapy regimens on survival, we compared survival among patients treated with IMRT alone, concurrent chemoradiotherapy, and adding neoadjuvant chemotherapy with DF regimen to IMRT and concurrent chemoradiotherapy (**Table 4**). Compared to IMRT alone, neoadjuvant with DF-concurrent chemoradiotherapyprovided a significantly better RFS and DFS (HR = 4.37; 95% CI, 1.37−14.00; P = 0.01for RFS and HR = 2.70; 95% CI, 1.16−6.30; P = 0.02 for DFS) and a similar OS and MFS (P = 0.10 and P = 0.31, respectively). There was no significant difference in the OS, MFS, RFS, and DFS between the patients receiving neoadjuvant chemotherapy with DF plus IMRT and the IMRT alone arm. Comparingneoadjuvant chemotherapy with miscellaneouschemotherapy regimens,neoadjuvant chemotherapy with DF regimen contributed to a similarsurvival benefit. However, a significant OS improvement was found favoring the addition of the neoadjuvant chemotherapy with DF regimen to concurrent chemoradiotherapy over concurrent chemoradiotherapy alone (HR = 13.37; 95% CI, 1.24−144.29; P = 0.03).

**Table 4:** Survival by treatment arm with DF regimen as neoadjuvant chemotherapy.

	Overall survival		Distant metastasis-freesurvival		Relapse-free survival		Disease-free survival	
	HR (95%CI)*	*P* *	HR (95%CI)*	*P* *	HR (95%CI)*	*P* *	HR (95%CI)*	*P* *
Neoadjuvant with DF-concurrent chemoradiotherapy vs IMRT alone	6.72 (0.70–64.14)	0.10	1.78 (0.58–5.45)	0.31	4.37 (1.37–14.00)	0.01	2.70 (1.16–6.30)	0.02
Neoadjuvent chemoradiotherapy with DF vs IMRT alone	0.27 (0.06–1.31)	0.10	1.98 (0.67–5.84)	0.21	1.63 (0.62–4.28)	0.32	1.58 (0.76–3.29)	0.22
Neoadjuvant with DF-concurrent chemoradiotherapy vs Concurrent chemoradiotherapy	13.37 (1.24–144.29)	0.03	1.92 (0.31– 11.77)	0.48	2.32 (0.60–8.99)	0.22	1.45 (0.56–3.70)	0.44

* Adjusted for age, gender, cigarette smoking, alcohol use, family history, histologic types, stage group, T stage and N stage in multivariate Cox model.

**Figure 2:**
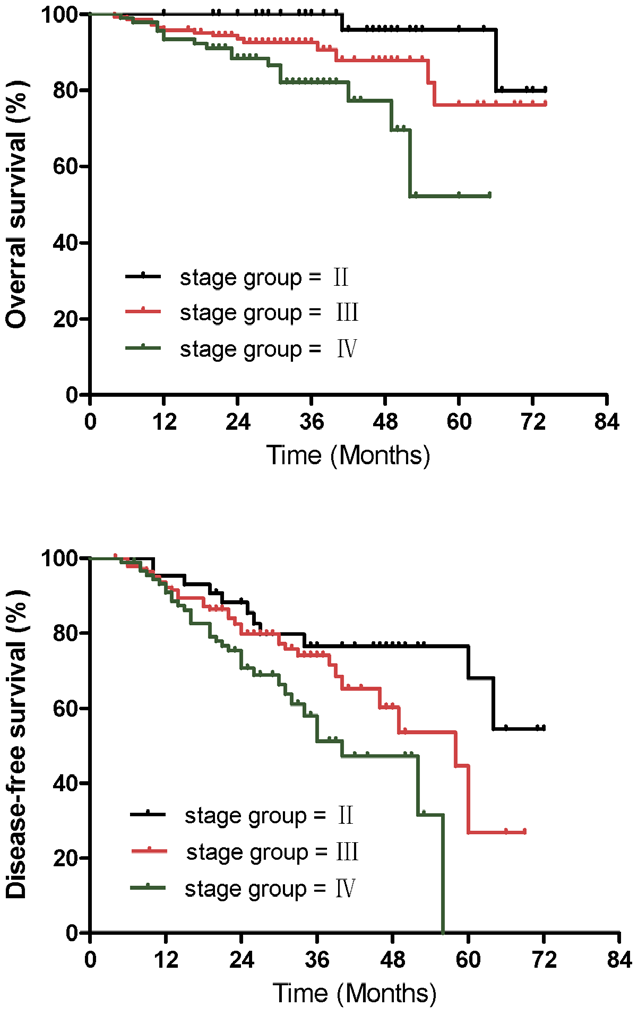
Kaplan-Meier estimates of Overral survival and disease-free survival for patients receiving with stage group being II versus III versus IV.

**Figure 3:**
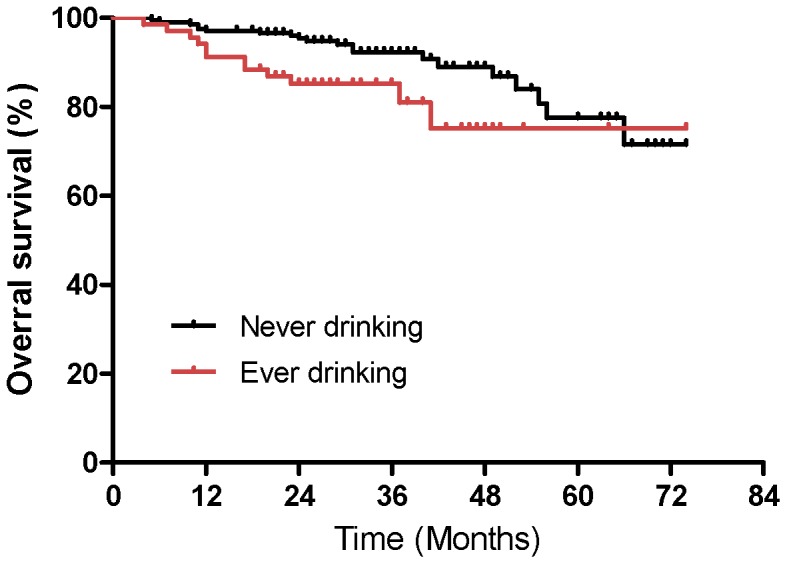
Kaplan-Meier estimates of overall survival of patients with alcohol drinking versus without alcohol drinking.

**Figure 4:**
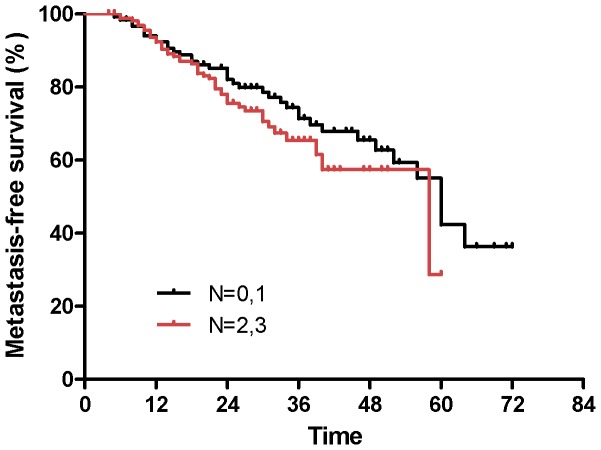
Kaplan-Meier estimates of metastasis-free survival for patients with N stage being 1 or 2 versus 3 or 4.

To identify which factors affected patient outcome, we performed univariate and multivariate analyses to evaluate the prognostic value of age, gender, cigarette smoking, alcohol use, family history, WHO histology, stage group, T stage, and N stage (**Table 5**). The results showed that compared to patients with stage II NPC, patients with stages IV and III were at an approximately 11- and 4-fold increased risk of death (HR = 11.05; 95% CI, 1.63−74.92; P = 0.01 for stage IV and HR = 4.54; 95% CI, 0.76−27.30; P = 0.09 for stage III; **Fig. 2**), respectively. Alcohol consumption was a negative prognostic factor for OS, with P values <0.001 based on univariate analysis (**Fig. 3**) and P = 0.06 based on multivariate analysis, respectively. Advanced N stage was associated with the occurrence of distant metastasis and MFS (HR = 2.79; 95% CI, 1.30−5.97; P = 0.02; **Fig. 4**). There was no significant association between the remaining predictors (age, gender, smoking status, family history of cancer, histology, and T stage) and the survival, recurrence, or metastasis rates based on univariate and multivariate analyses.

**Table 5:** Effect of prognostic factors on survival in multivariable analyses.

	Overall survival		Distant metastasis-freesurvival		Relapse-free survival		Disease-free survival		
	HR (95%CI)*	*P* *	HR (95%CI)*	*P* *	HR (95%CI)*	*P* *	HR (95%CI)*		*P* *
Gender, Female vs Male	0.94 (0.38–2.34)	0.89	1.24 (0.56−2.76)	0.60	0.90 (0.44–1.80)	0.76	0.95 (0.55−1.65)		0.87
Age, >50y vs ≤50 y	0.88 (0.42– 1.85)	0.74	1.30 (0.69−2.47)	0.42	0.87 (0.48−1.57)	0.64	1.11 (0.70−1.75)		0.66
Smoking status, Ever vs Never	1.05 (0.44–2.49)	0.91	0.93 (0.45−1.93)	0.84	1.32 (0.69−2.55)	0.41	1.08 (0.64−1.81)		0.77
Drinking status, Ever vs Never	2.17 (0.96−4.91)	0.06	1.74 (0.85–3.56)	0.13	0.88 (0.43−1.80)	0.73	1.09 (0.63−1.88)		0.75
Stage group, III vs II	4.54 (0.76–27.30)	0.09	1.62 (0.29−9.05)	0.58	1.06 (0.33−3.40)	0.92	1.05 (0.40−2.73)		0.92
Stage group, IV vs II	11.05 (1.63–74.92)	0.01	2.91 (0.48−17.82)	0.25	1.44 (0.40−5.21)	0.58	1.69 (0.59−4.83)		0.33
Family history, Yes vs No	1.48 (0.64–3.45)	0.36	0.95 (0.43−2.12)	0.91	0.92 (0.43−2.00)	0.84	0.92 (0.51–1.66)		0.77
Histology, 2 vs 1	1.93 (0.41–9.02)	0.40	1.33 (0.38–4.66)	0.66	0.75 (0.33–1.73)	0.50	0.87 (0.42–1.79)		0.70
Histology, 3vs 1	1.57 (0.33–7.53)	0.57	1.45 (0.40–5.29)	0.57	0.58 (0.24–1.44)	0.24	0.74 (0.35–1.60)		0.45
T stage, T3–T4 vs T1–T2	0.71 (0.25–2.02)	0.52	1.82 (0.74–4.49)	0.20	1.44 (0.57–3.64)	0.44	1.64 (0.83–3.24)		0.16
N stage, N2–N3 vs N0–N1	0.83 (0.34–2.00)	0.67	2.79 (1.30–5.97)	0.01	0.85 (0.44–1.67)	0.64	0.16		

* Adjusted for age, gender, cigarette smoking, alcohol use, family history, histologic types, stage group, T stage and N stage in multivariate Cox mode.

## Discussion

IMRT has yielded remarkable advances compared to conventional radiotherapy in target conformity, increased radiation dose in the target volume, and sparing of surrounding normal organs at risk [14], [15]. The utilization of IMRT is particularly valuable in patients with NPC because the nasopharynx area is complex and surrounded by a number of critical normal structures, including the brain, brain stem, spinal cord, optic pathway, middle and inner ear, parotid glands, and temporomadibular joint, and IMRT yields a concave dose distribution to avoid organs at risk and thus permit the irradiation of patients with complex-shaped tumors in locations which are difficult to access. The clinical applications of IMRT in patients with NPC demonstrated that this radiation technique could improve tumor control [5], [7], reduce locoregional and regional failure, provide better survival and quality of life [16], and significantly enhance the therapeutic ratio. In addition, IMRT could maximize protection of normal structures and reduce and delay the onset of acute toxicity [17]. Consequently, IMRT is widely used in treating NPC and has been adopted as the standard of care in many centers that treat patients with NPC.

Conventional radiotherapy combined with chemotherapy was formerly the standard treatment modality for locoregionally advanced NPC [18], [19]. The Intergroup-0099 Study first revealed that the addition of cisplatin-based concurrent and adjuvant chemotherapy to conventional radiotherapy could contribute to improvement of event-free survival and OS [9]. Subsequent randomized trials confirmed the survival benefit provided by adding chemotherapy to conventional radiotherapy [20], [21]. In clinical practice, cisplatin-based chemotherapy in concurrence with conventional radiotherapy has also proved its superiority to conventional radiotherapy alone for the treatment of locoregionally advanced NPC [21]. However, the contribution of adding chemotherapy to radiation has not been confirmed when conventional radiotherapy is replaced with IMRT [10], [11]. No significant survival benefit with chemotherapy over IMRT alone was demonstrated primarily by achieving a higher local tumor control rate or less metastasis or both in locoregionally advanced NPC patients.

In the current retrospective cohort study, we first reported significant improvement of RFS and borderline significantly elevated DFS favoring the IMRT plus chemotherapy arm for patients with locoregionally advanced NPC in the Cox model adjusted for the prognostic factors. Further analyses demonstrated that the survival improvement of RFS and DFS were indeed from the addition of concurrent chemotherapy and neoadjuvant-concurrent chemotherapy, not from the addition of neoadjuvant chemotherapy. Our findings confirmed the survival benefit with concurrent chemotherapy-based treatment over radiation alone when conventional radiotherapy is replaced with IMRT, although it is inconsistent with the previous comparison between IMRT with and without chemotherapy. There are only three retrospective studies comparing the survival of IMRT alone and IMRT combined with chemotherapy [10], [11], [12]. A study conducted in China and Singapore observed 107 patients with stage IIb NPC to compare IMRT alone and IMRT combined with various strategies of chemotherapy, including neoadjuvant, concurrent and adjuvant chemotherapy, and found no significant difference in disease control and survival rates in patients treated with or without chemotherapy of any schedule [11]. Another retrospective study assigned 370 patients with locoregionally advanced NPC to compare IMRT with or without concurrent chemotherapy and revealed no significant improvement in local control and rates of OS, MFS, and DFS [10]. However, in the current study adjuvant chemotherapy and neoadjuvant chemotherapy were applied in patients at the discretion of radiation oncologists, which could confound the contribution of the concurrent chemotherapy to IMRT [10]. Su et al.^12^ also demonstrated that patients with locoregionally advanced NPC had similar OS, MFS, and DFS when treated by IMRT-based modalities, including IMRT alone, chemoradiotherapy, adjuvant chemoradiotherapy, concurrent chemoradiotherapy, neoadjuvant-concurrent chemoradiotherapy, and concurrent-adjuvant chemoradiotherapy. Nevertheless, in their studies, patients received treatment based on the selection criteria that patients with poor conditions would be treated by IMRT alone and patients with more advanced N stage would receive more chemotherapy. Therefore, we presume that the reason for failing to find a significant improvement in survival with chemotherapy over IMRT alone in previous studies could probably be attributed to the early cancer stage of patients, confounding of other treatment regimens, and treatment selection to patients, thus the addition of chemotherapy to IMRT might contribute to a significant improvement in survival in a well-designed study. Our presumption, which is partly supported by the previous study conducted by Su et al. [12] in which patients with a more advanced N stage because of receiving additional chemotherapy, had similar treatment outcomes to patients with a less advanced N stage who received less chemotherapy. Nevertheless, to validate our presumption regarding the efficacy of chemotherapy and the best strategy for combined treatment, larger, well-designed studies with balanced prognostic factors should be conducted in other patient populations with locoregionally advanced NPC.

Indeed, our findings are biologically plausible. First, cisplatin can enhance radiation-induced cell killing and sensitize the radiation [22], [23], [24], and it has been verified that radiosensitivity in head and neck cancer can be enhanced by chemotherapy *in vivo* [25], which is why concurrent, but not neoadjuvant chemotherapy, can contribute to the improvement in survival. Second, NPC is not only radiosensitive, but also chemosensitive [6], [9]. Chemotherapy should be able to improve survival through decreasing the tumor burden, eradicating distant micrometastases, and reducing disease failure. Finally, chemotherapy could reduce hypoxia in the primary site and metastatic lymph nodes by shrinking the tumor size, which could increase radiosensitivity and increase disease control [26], [27].

We also first explored the prognostic value of some carcinogenic factors, including cigarette smoking, alcohol use, and family history of cancer, and showed that alcohol use was a prognostic factor predictive of OS. The underlying biological mechanism of how alcohol use influences the association of survival with the treatment strategy in NPC patients is not clear. We speculate that alcohol consumption, as a risk factor for the etiology of NPC [28], has been reported to influence the histologic distribution of NPC [29], [30], [31] and thereby impact the prognosis. Another reason for alcohol use associated with poor prognosis might be that regular alcohol use will lead to poor liver function and elevated LDH, which are negative prognosticators for OS, DFS, and DMFS for NPC patients [32]. Nevertheless, to confirm the prognostic value for patients with locoregionally advanced NPC, larger well-designed clinical trials should be conducted in different populations and involve more risk factors to adjust the association.

As a retrospective analysis, the current study had limitations. First, because patients were only included in our study when the selection criteria were met, a selection bias might have occurred. However, such a selection bias most likely has a minimal impact on our result because only a small proportion of patients were excluded and there was still a large study population for the present analysis. Second, the current study was a non-randomized cohort comparison, which might have resulted in selection bias and imbalance between the two groups. Nevertheless, in this study the groups were shown to be well-matched for prognostic factors, except N stage. By contrast, the combined treatment group had more patients with advanced disease, and might have resulted in a survival benefit gained from the combined modality of treatment underestimated by the log-rank test. However, after adjustment with prognostic factors, the potential impact from confounding factors on the hazard ratio might be minimized. Another possible source of bias in the current study might result from the insufficient follow-up and small number of patients studied, which may result in an inadequate number of events needed for analysis and limit the assessment of long-term results of combined modality therapy. Therefore, larger studies with an extended follow-up are needed to confirm these findings in patients with NPC.

In summary, our study provides evidence that IMRT combined with concurrent chemotherapy or neoadjuvant-concurrent chemoradiotherapy resulted in an improvement in DFS or RFS for patients with locoregionally advantage NPC compared to IMRT alone. In addition, our study was the first to determine the prognostic value of some carcinogenic factors, such as smoking status, alcohol use, and family history of cancer, and showed that alcohol consumption was a prognostic factor for OS. This study could be of value to guide effective individualized treatmen for patients with NPC and may be helpful to guide effective individualized treatment. In an attempt to further reveal the contribution of chemotherapy to IMRT and evaluate the long-term results of combined modality therapy, we will recruit more patients to our program and lengthen the follow-up in the future.
